# Sugar Transporter ZjSWEET2.2 Mediates Sugar Loading in Leaves of *Ziziphus jujuba* Mill

**DOI:** 10.3389/fpls.2020.01081

**Published:** 2020-07-24

**Authors:** Yanqiu Geng, Mengjia Wu, Chunmei Zhang

**Affiliations:** State Forestry and Grassland Administration Key Laboratory of Silviculture in Downstream areas of the Yellow River, College of Forestry, Shandong Agricultural University, Tai’an, China

**Keywords:** Jujube (*Ziziphus jujuba* Mill.), SWEET, photosynthesis, sugar signal, sugar transporter

## Abstract

In plants, sugar transporters play an important role in the allocation of sugars from cells in source organs to cells in sink organs. Hence, an understanding of the molecular basis and regulation of assimilate partitioning by sugar transporters is essential. Leaves are the main source of photosynthetic products. In jujube (*Ziziphus jujuba* Mill.), the mechanisms regulating initial sugar unloading in leaves are still unclear. In this study, an expression profiling analysis showed that *ZjSWEET2.2*, encoding a sugar transporter in the SWEET family, is highly expressed in leaves. Over-expression of *ZjSWEET2.2* increased carbon fixation in photosynthetic organs. Our analyses showed that *ZjSWEET2.2* encodes a plasma membrane-localized sugar transporter protein. Its expression levels were found to be suppressed under drought stress and by high concentrations of exogenous sugars, but increased by low concentrations of exogenous sugars. Finally, DNA sequence analyses revealed several *cis*-elements related to sugar signaling in the promoter of *ZjSWEET2.2*. Together, these results suggest that ZjSWEET2.2 functions to mediate photosynthesis by exporting sugars from photosynthetic cells in the leaves, and its gene expression is regulated by sugar signals.

## Introduction

In higher plants, photosynthates produced at the photosynthetic “source” (mainly mesophyll cells) are essential for growth and development, and are major nutritional components of fruits (Zhu et al., 2010). Photosynthate allocation occurs *via* transport from the source organ to the heterotrophic “sink” organs (fruits, roots, seeds) (Lalonde et al., 2004; Ayre, 2011). Adequate photosynthate production in the leaves can substantially increase the number of flowers and fruit weight. Improvements in allocation efficiency can increase the proportion of total biomass allocated to harvestable organs (Zhu et al., 2010; Paterson and Li, 2011). Sugar translocation depends on active transport by transmembrane proteins or passive transport *via* the plasmodesmata. In higher plants, sugar transporters play crucial roles in mediating carbohydrate fluxes (Patrick et al., 2001) and are linked with biomass gain. By now, sugar transporters found in plants mainly include three types: monosaccharide transporters (MST), sucrose transporters (SUT) and SWEET transporters (Sugars Will Eventually be Exported Transporters). Of these, MSTs and SUTs have typical structural characteristics of the major facilitator superfamily (MFS) with high hydrophobicity and generally include 12 transmembrane domains (Fang et al., 2020), which is the most important membrane transporter family. SUTs are crucial for the sucrose long-distance transportation in phloem to sink organs as sucrose is the important form of photosynthetic products (Chandran et al., 2003; Scofield et al., 2007). In the sink organs, some of the disaccharide can be degraded to monosaccharide and mediated by MSTs (Doidy et al., 2019), while in pea seeds, most carbohydrates transported into cotyledons cells in the form of sucrose by sucrose transporter *PsSUT1* (Zhou et al., 2009). Besides, sucrose can be used as an important sugar signal to regulate plant growth and development (Osuna et al., 2007).

As a novel family of sugar transporters, the role of SWEET proteins was first identified in *Arabidopsis thaliana* (Chen et al., 2010). A protein structure prediction analysis indicated that SWEET transporter protein belonged to the MtN3 family and have seven transmembrane domains (TMSs) with two duplicated units of three TMSs and a linker element (the fourth TMS) (Chen et al., 2012; Xuan et al., 2013). SWEET proteins have sugar efflux and influx activity, and have diverse physiological functions (Jeena et al., 2019). Other studies sequenced and characterized SWEET family members in various plant species, such as tomato (*Solanum* lycopersicum), wheat, citrus, and pear (*Pyrus* *bretschneideri*) (Xu et al., 2013; Feng et al., 2015; Li et al., 2017; Gao et al., 2018). The results of those studies provided some details of the roles of SWEET transporters in regulating sugar transport and accumulation. SWEET transporters show low affinity for hexose and sucrose during sugar efflux or import. In *Nicotiana, Arabidopsis*, and brassicas, SWEET9 facilitates sucrose efflux for nectar secretion (Lin et al., 2014). SWEET15 mediates sugar export in the endosperm to promote embryo development (Wang et al., 2019b) or seed filling (Chen et al., 2015a). Other SWEET proteins function under different osmotic stress conditions or in pathogenic reactions (Chen et al., 2015b; Jeena et al., 2019). Some SWEET proteins are involved in the mobilization of carbohydrates in leaves (Chen et al., 2010), which may affect photosynthetic efficiency. In *Arabidopsis*, AtSWEET11 and 12 were shown to play crucial roles in sugar efflux from mesophyll cells to the apoplast (Chen et al., 2012); and in tomato, SlSWEET1a was found to play roles in glucose efflux from mature to young leaves (Ho et al., 2019). Nevertheless, the function of many SWEET sugar transporters, especially in perennial woody crops, remains unclear.

Jujube (*Ziziphus jujuba* Mill.), a member of the Rhamnaceae family, is an important dry fruit crop with a worldwide distribution (Qu and Wang, 1993). It is strongly resistant to drought and salinity stress. With a cultivation area of more than 2 million ha, jujube has become the primary source of income for 20 million farmers. Our previous studies identified that sugar transporters play significant roles in the accumulation of sugars in jujube fruit (Zhang et al., 2018). However, little is known about the mechanism of sugar efflux by sugar transporters in leaves. In this study, we identified ZjWEET2.2 as another member of the SWEET family in Z. *jujuba*, and conducted a preliminary evaluation of its functions. The gene encoding ZjWEET2.2 was found to be highly expressed in photosynthetic organs, and the protein was found to localize to the plasma membrane. Over-expression of this gene increased carbon fixation. Our results indicate that the expression of *ZjSWEET2.2* and the activity of its encoded protein are mediated by sugar signals. The results of this study provide new insights into the function and regulation of SWEET sugar transporters.

## Materials and Methods

### Plant Materials

The Chinese dry jujube cultivar “No. 4 Jinsi,” and fresh jujube “Zaozhuangcuizao” and “Dongzao,” were grown at the Jujube Experimental Station, Shandong Institute of Pomology, Taian, China. The fruits of “No. 4 Jinsi” were collected at different developmental stages [young fruit at 10 days after anthesis (DAA), enlarged fruit at 40 DAA, white mature fruit at 80 DAA, half-red fruit at 100 DAA, and fully red fruit at 110 DAA]. Leaf, root, and phloem (scraped from the stem using a blade) samples were also collected.

Jujube young trees were grown in an incubator under a 16-h light/8-h dark photoperiod, and watered every five days to keep water content at more than 60% of maximum field capacity. For drought stress treatments, water was withheld for 2 weeks when the leaves showed wilting phenotype; while the control group was keep watering every five days. The leaves were harvested from water deficit-treated trees and well watered controls for analyses. In the exogenous sugar application experiment, the treatment groups were sprayed with 3% or 10% (w/v) glucose or sucrose, and the control group was sprayed with the same amount of distilled water. Leaves were collected at 6 h after treatment for analyses. For each treatment, three biological replicates were analyzed. All samples were directly frozen in liquid nitrogen and stored at −80°C until analysis.

### Construction of Phylogenetic Tree

Jujube genes putatively encoding sugar transporter proteins were obtained from the Junzao genome (accession number: LPXJ00000000) (Huang et al., 2016). The BLASTP tool was used to retrieve loci encoding putative sugar transporters, using *Arabidopsis* sequences as queries with E-values ≤1^e-5^. To construct the phylogenetic tree, the sequences of transporters from jujube, *Arabidopsis*, and *Solanum lycopersicum* L. were aligned by ClustalW with MEGA5 (Tamura et al., 2011). The phylogenetic tree was constructed using the Neighbor-Joining method, and the phylogeny test was based on the bootstrap method with 1000 replications.

### Vector Construction and Plant Transformation

The vector was constructed using the method described by Jiang et al. (2017b). The coding sequence of *ZjSWEET2.2* was inserted downstream of the CaMV35S promoter in the plant expression vector pVBG23000-GFP, which includes the gene encoding green fluorescent protein (GFP). For the subcellular localization analysis, the positive vector CaMV35S-*ZjSWEET2.2*-GFP was transiently expressed in tobacco leaves after infiltration with *Agrobacterium* *tumefaciens* strain GV3101 containing the vector. The pRT101-AtPIP2A-red fluorescent protein (RFP) was used as a plasma membrane marker, and was co-transformed with CaMV35S-ZjSWEET2.2-GFP. The tobacco plants were incubated at 25°C for 48 to 72 h, and the fluorescence of GFP was observed under a high resolution laser confocal microscope (Zeiss, Jena, Germany).

For the transient overexpression analysis, jujube leaves were vacuum-infiltrated with *Agrobacterium* containing the vector along with 500 µl Tween 20. The CaMV35S-GFP construct was used as a control. The leaves were cultured on Murashige and Skoog (MS) medium for 48 to 72 h in an incubator under a 12-h dark/12-h light photoperiod. The presence and relative amount of the transgene in transgenic leaves were determined by qRT-PCR.

### Analyses of mRNA Levels

Total RNA was extracted using a Plant RNA Extraction Kit (Foregene, Chengdu, China) following the manufacturer’s instructions. To analyze the transcript levels of *ZjSWEET2.2* and genes related to carbon fixation [*RBCS* (encoding ribulose bisphosphate carboxylase/oxygenase), *PGK* (encoding phosphoglycerate kinase), *RPI* (encoding ribose-5-phosphate isomerase), and *PRK* (encoding phosphoribulokinase)], reverse-transcription quantitative real-time PCR (qRT-PCR) was performed using a SYBR Premix Ex Taq Kit (Vazyme, Nanjing, China) on a Bio-Rad IQ5 instrument (Bio-Rad, Hercules, CA, USA). The reaction volume was 20 µl. All qRT-PCRs were performed with three technical replicates and three biological replicates. The primers were designed from the coding sequences of jujube genes using Primer5 software and are listed in **Supplemental Table 1**. Primer specificity was determined based on melting curve analyses. Expression data were analyzed using the 2-^ΔΔCT^ method (Livak & Schmittgen, 2001). The selected reference gene was *UBQ* (Zhang et al., 2015).

### Measurement of Sugar Contents

The contents of sucrose, glucose, and fructose were measured by high performance liquid chromatography (HPLC) according to Gao and Wang, (2013). All samples were extracted and analyzed in triplicate. For each sample, 1 g tissue was homogenized and measured. Three biological replicates were analyzed for each time point and variety.

## Results

### Sequence Analysis of *ZjSWEET2.2*

**Figure 1:**
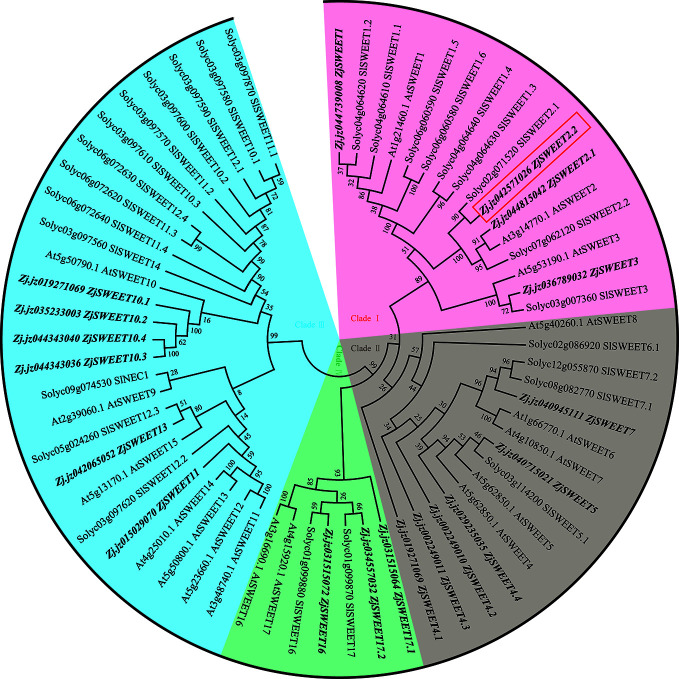
Maximum likelihood phylogeny of SWEET sugar transporters. Accession numbers for SWEET genes from *Arabidopsis* are those reported by Wei et al. (2014) and those for tomato genes are those reported by Feng et al. (2015).

We identified 19 candidate *ZjSWEET* genes from the jujube genome. The phylogenetic tree was constructed using amino acid sequences of SWEET transporters from jujube, *A. thaliana*, and *S. lycopersicum* (**Supplemental Table 2**). The SWEET sugar transporters from the three species could be classified into four clades (**Figure 1**). Clade I includes the members of SWEET1, SWEET2, and SWEET3, subclades), clade II includes the members from SWEET4 to SWEET8, clade III includes the members from SWEET9 to SWEET 15, and clade IV includes SWEET16 and SWEET17. Consistent with the fact that a recent common whole genome duplication event has occurred in many species, there were duplicate copies of some *SWEET* genes in the genome. In our comparisons, *ZjSWEET2.2* in clade I of the SWEET family showed the closest relationship with its paralog *ZjSWEET2.1* and its ortholog *AtSWEET2* from *A. thaliana*. Protein structure analyses of ZjSWEET2.2 revealed seven predicted TMDs in a protein comprising 266 amino acids (**Supplemental Figure 1**).

### Subcellular Localization of *ZjSWEET2.2*

**Figure 2:**
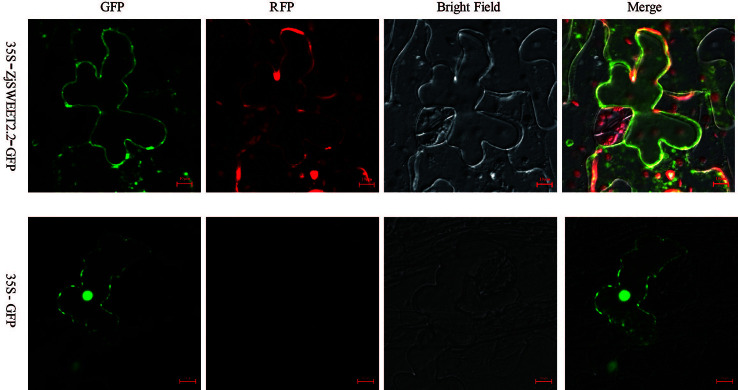
Subcellular localization of ZjSWEET2.2. 35S:GFP-*ZjSWEET2.2* was transiently expressed in tobacco leaves and co-localized with transiently expressed plasma membrane marker RFP. Scale bars, 10 μm.

The coding sequence for *ZjSWEET2.2* was fused to eGFP and transiently expressed in leaves of *Nicotiana benthamiana* to determine the subcellular localization of its encoded protein. The construct was co-expressed with the mRFP1-labeled plasma membrane marker. Confocal images showed that GFP fluorescence was localized to the plasma membrane, the cytoplasm and nucleus of the tobacco epidermal cells, and ZjSWEET2.2-GFP was co-located with the plasma membrane marker at the plasma membrane (**Figure 2**).

### Tissue Expression Patterns of *ZjSWEET2.2*

**Figure 3:**
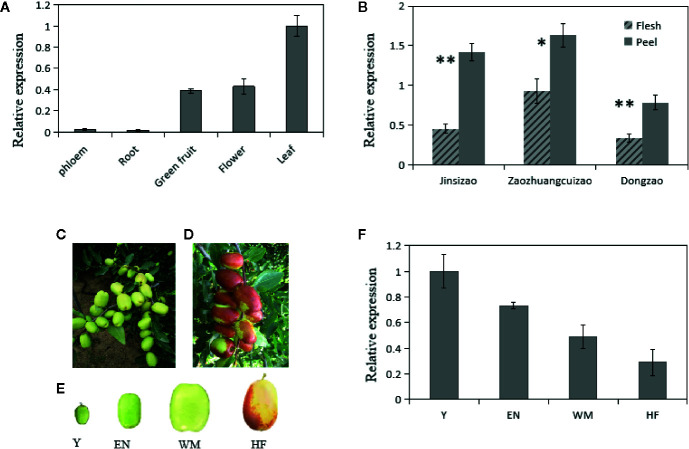
Transcript levels of *ZjSWEET2.2* in different tissues of jujube and phenotypic characteristics of fruits at different development stages. **(A)** Transcript levels of ZjSWEET2.2 in phloem, root, flowers, leaves, and fruit. **(B)** Transcript levels of ZjSWEET2.2 in peel and flesh of three different jujube varieties. **(C)** White mature fruit and **(D)** half-red fruit on jujube tree. **(E)** Four different development stages of jujube fruits (Y, young fruit; EN, enlargement stage; WH, white mature stage; HF, half-red stage). **(F)** Changes in relative transcript levels of ZjSWEET2.2 during fruit development. Three biological replicates were analyzed. Error bars represent SE. Asterisks indicate significant difference as determined by Student’s t-test (**P < 0.01; *P < 0.05).

Our previous RNA sequencing results showed that *ZjSWEET2.2* transcript levels were highest in leaves among various tested jujube tissues (spires, leaves, flowers, fruit, and stems) (Huang et al., 2016; PRJNA306374). To explore the putative functions of *ZjSWEET2.2*, we determined its transcript levels in different tissues (**Figure 3**). The transcript levels of *ZjSWEET2.2* were highest in the leaves followed by flowers and green fruit, while there were low transcript levels in the root and the phloem of branches (**Figure 3A**). We also determined its transcript levels in the flesh and green peel of three different cultivars (**Figure 3B**). In all cultivars, the *ZjSWEET2.2* transcript levels were higher in the peel than in the flesh, consistent with the higher chlorophyll content in the peel than in the flesh. Furthermore, *ZjSWEET2.2* transcript levels were analyzed in different development stages of fruit (peel) (**Figure 3F**). The transcript levels of *ZjSWEET2.2* in fruit were high at the young stage and decreased as fruit ripened, alongside the degradation of chlorophyll.

### Overexpression of *ZjSWEET2.2* in Transgenic Lines

**Figure 4:**
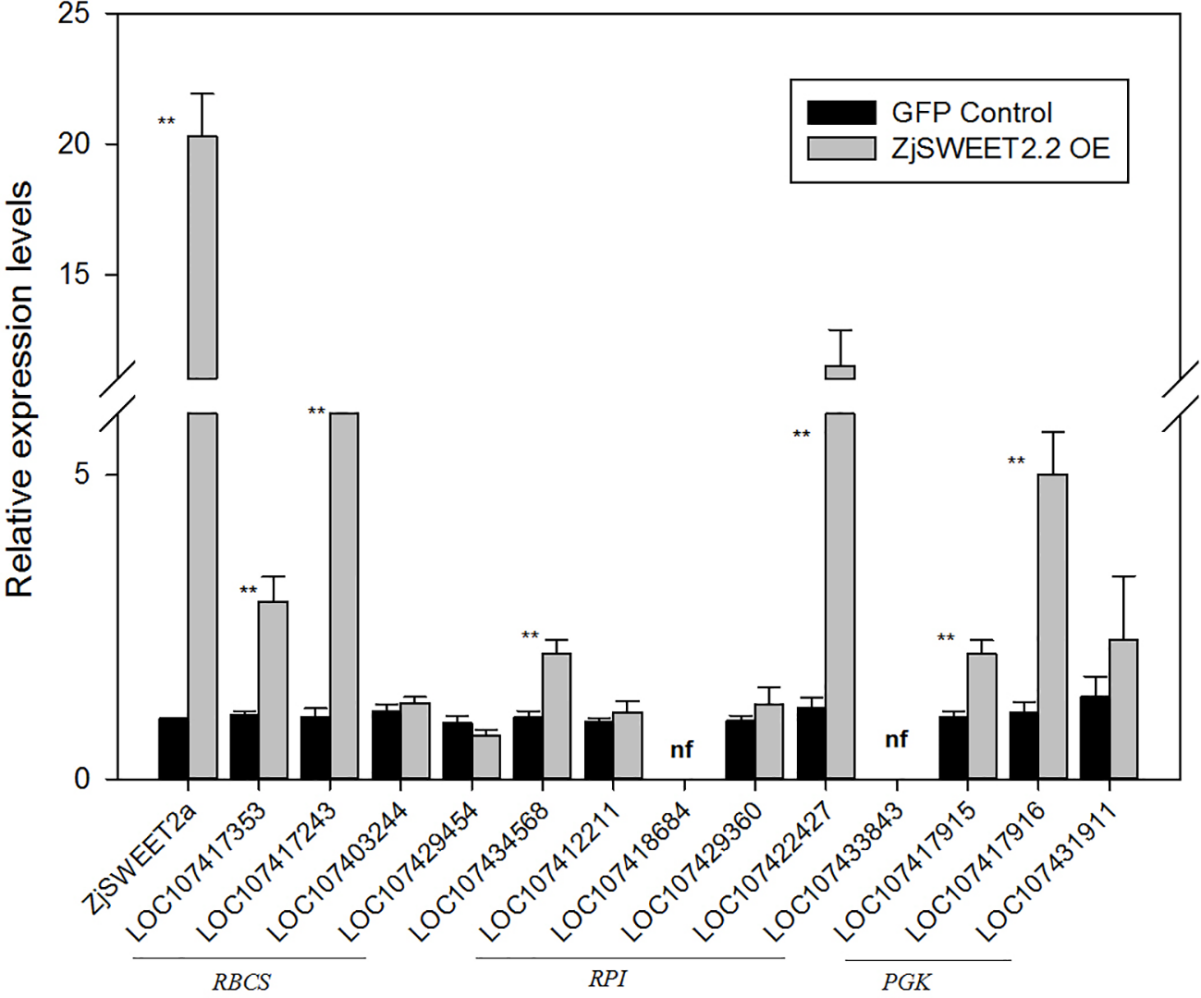
Transcript levels of genes involved in carbon fixation in photosynthetic organ. RBCSs: ribulose bisphosphate carboxylases; PRKs, phosphoribulokinases, RPIs: ribose-5-phosphate isomerases, PGKs: phosphoglycerate kinases. Results are normalized against transcript level in control group, which was set to 1. Data are mean ± SD of three replicates. ** indicate significant difference (P < 0.01) as determined by Student's t-test.

To explore the patterns of regulation of *ZjSWEET2.2* in leaves, the CaMV 35S: ZjSWEET2a-GFP construct was transformed into jujube leaves for transient expression. We then determined the transcript levels of *ZjSWEET2.2* and genes related to photosynthetic carbon assimilation by qRT-PCR. The transcript level of *ZjSWEET2.2* in the ZjSWEET2.2-OE line was nearly 20 times higher than that in the GFP-expressing control. We determined the transcript levels of genes encoding RBCs, the key enzyme in carbon fixation in the leaf; RPIs, which function upstream of RBCs; and PGKs, which function downstream of this pathway (**Figure 4**). Two genes in the RBCs family were significantly up-regulated in the ZjSWEET2.2-OE line compared with the control. Among the RPIs family, two members were expressed weakly and two showed no differences in their transcript levels between the ZjSWEET2.2-OE line and the control. However, the LOC107422427 transcript levels in leaves were higher in *ZjSWEET2.2*-OE than in the control. One *PRK* gene was up-regulated in the ZjSWEET2.2-OE line. In the *PGKs* gene family, *PGK1* (LOC107431911) transcript levels were not affected in the ZjSWEET2.2-OE line, but the other two *PGKs* were up-regulated. Together, these results suggested that overexpression of *ZjSWEET2.2* increased carbon fixation to some extent *via* increased export of assimilation products from photosynthetic tissues.

### Regulation of *ZjSWEET2.2* by Sugars

**Figure 5:**
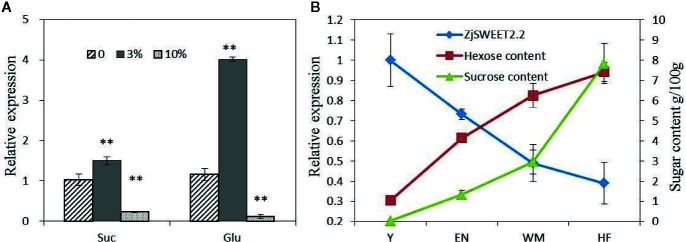
Transcript levels of *ZjSWEET2.2* in leaves of plants treated with exogenous sugars **(A)**, and correlation between transcript levels of *ZjSWEET2.2* and monosaccharide sugar content **(B)**. Three biological replicates were analyzed. Error bars represent SE. ** indicate significant difference (P < 0.01) as determined by Student’s t-test.

Exogenous sugars at two concentrations were sprayed onto the leaves of jujube plants, and then the transcript levels of *ZjSWEET2.2* were monitored by qRT-PCR (**Figure 5A**). After treatment with 3% (w/v) exogenous glucose, the transcript level of *ZjSWEET2.2* was significantly up-regulated by approximately three-fold, compared with that in the control. In the plants treated with 3% (w/v) exogenous sucrose, the transcript level was also increased, but only by 50% compared with that in the control. When the concentration of exogenous sugars was increased to 10% (w/v), the transcript level of *ZjSWEET2.2* decreased to lower than that in the control, and this difference was more significant in the plants treated with glucose than in those treated with sucrose. These results showed that the expression of *ZjSWEET2.2* was induced by low concentrations of sugars, but suppressed by high concentrations of sugars, and was more responsive to glucose than to sucrose.

Next, we analyzed the correlation between the transcript levels of *ZjSWEET2.2* and sugar contents in fruit during development. The contents of hexoses (fructose and glucose) increased steadily during fruit development, while sucrose began to accumulate rapidly from the white ripening stage (2.9 g/100 g fruit fresh weight), ultimately reaching 7.8 g/100 g (**Figure 5B**). A correlation analysis showed that the transcript level of *ZjSWEET2.2* was significantly negatively correlated with hexose content (r = −0.985, *P* < 0.015).

To explore the regulation of *ZjSWEET2.2* expression, the 2-kb promoter region upstream (5′) of the start codon of *ZjSWEET2.2* was sequenced and analyzed (**Supplemental Figure 2**). Tools at the PlantCare server were used to identify *cis*-elements in this sequence, and additional *cis*-elements that are known to be sugar regulation regions were also considered (Li et al., 2006; Baena-Gonzalez et al., 2007). In total, 12 *cis-*elements related to sugar repression and seven related to sugar induction were identified (**Table 1**). Interestingly, most of these elements were located within the 1-kb region upstream of the start codon. The large number of sugar signaling-related *cis-*elements in close proximity to the start codon strongly suggested that sugar signals regulate the expression of *ZjSWEET2.2*.

**Table 1:** Known cis-acting elements involved in sugar repression/induction in 2,000-bp fragment of ZjSWEET2.2 promoter.

Cis-Elements	Sequence	Response	Copies	References
W-box	TTGACC	Sugar induce	2	Sakr et al., 2018; Chen et al., 2019
W-box	TGACT	Sugar induce	1	Sakr et al., 2018
SUCROSE BOX 3	AAATCA.AA	Sugar induce	4	Hwang et al., 1998; Lu et al., 1998;
TATCCAOSAMY	TATCCA	Sugar suppress	2	Lu et al., 2002; Sun et al., 2003 Baena-Gonzalez et al., 2007
G box	CACGTG	Sugar suppress	1	Hwang et al., 1998
CATCC	CATCC	Sugar suppress	2	Li et al., 2006
I-BOX core	GATAA	Sugar suppress	3	Manzara et al., 1991
AMYBOX1	TAACAAA	Sugar suppress	1	Hwang et al., 1998
EVENINGAT core	ATATCT	Sugar suppress	2	Li et al., 2006
GATTA	GATTA	Sugar suppress	1	Li et al., 2006

### Suppression of *ZjSWEET2.2* Expression Under Drought Stress

We determined the transcript levels of *ZjSWEET2.2* in leaves of jujube plants under drought stress. The results showed that the transcript level decreased to approximately one-quarter its pre-stress level under drought stress, suggesting that SWEET2 plays important roles in regulating cell osmotic potential by reversing sugar efflux. Consistent with this, the contents of fructose and glucose in leaves of jujube increased under drought stress (**Supplemental Figure 3**).

## Discussion

### *ZjSWEET2.2* Plays Roles in Mediating Sugar Export From Photosynthetic Organs

The SWEET sugar transporters from the three species could be classified into four clades, previously uncovered in Arabidopsis (Chen et al., 2010; Eom et al., 2015; Doidy et al., 2019). Our analyses showed that *ZjSWEET2.2* is in the clade I *SWEET* gene family, and is phylogenetically closest to *AtSWEET2*. In *Arabidopsis*, *AtSWEET2* encodes a glucose transporter (Xuan et al., 2013; Chen et al., 2015a). In this study, we found that *ZjSWEET2.2* could be regulated by exogenous glucose, but its expression was relatively insensitive to exogenous sucrose. Therefore, we speculate that ZjSWEET2.2 also mainly exports glucose. We detected low transcript levels of *ZjSWEET2.2* in sink organs of jujube, but high transcript levels in leaves, where photosynthates are produced and then continuously exported to sink organs. Although sucrose may account for the largest proportion of sugars exported from the leaf, it can also be hydrolyzed into glucose and fructose by cell wall invertases, thereby forming a sucrose gradient to facilitate outflow (Osorio et al., 2014). When the ZjSWEET2.2 protein was transiently expressed in tobacco leaves, it co-localized with a plasma membrane marker in the plasma membrane, suggesting that this is the primary site of apoplastic sugar transport. On the basis of our results, we suggest that the plasma membrane-localized sugar transporter ZjSWEET2.2 directly transports glucose and indirectly facilitates sucrose loading in the leaf. AtSWEET2 limits carbon sequestration in the roots in *Arabidopsis*, while LcSWEET2a in *Litchi chinensis* is involved in early seed development (Xie et al., 2019). In contrast, *ZjSWEET2.2* is expressed abundantly in chlorenchyma cells. Besides, AtSWEET2 was characterized as a vacuolar transporter (Chen et al., 2015b), while the orthologous gene ZjSWEET2.2 is located at the plasma membrane. We speculate that the function of SWEET2 may differ among different species. Soluble sugars are known to be involved in the regulation of osmotic potential in cells of plants under salt and drought stress (Lemoine et al., 2013). In our study, the transcript level of *ZjSWEET2.2* significantly decreased in jujube plants under drought stress. This led to decreased sugar export from cells, so that the content of hexose sugars increased. Together, our results suggest that *ZjSWEET2.2* plays a key role in sugar export from source (photosynthetic) leaves.

Previous studies have shown that the over-accumulation of carbohydrates in leaves reduces the photosynthetic rate (Baker et al., 2016). In *Arabidopsis*, ATSWEET11 and 12 are located in the vascular tissues of leaves. Mutations of their encoding genes were shown to result in the accumulation of starch in leaves, which seriously reduced photosynthetic efficiency (Chen et al., 2012). Similar phenomena have been observed in *Zea mays*, in which photosynthesis was impaired in the genome-edited knock-out mutants of ZmSWEET13 paralogs (a, b, and c) (Bezrutczyk et al., 2018). In contrast, increasing sugar efflux can stimulate photosynthetic activity (Ainsworth and Bush, 2011). The maize protein CTS1, a homolog of ATSWEET1, increases photosynthesis by regulating the sugar content in subsidiary cells in maize (Wang et al., 2019a). In our study, overexpression of *ZjSWEET2.2* resulted in increased transcript levels of genes related to carbon fixation and photosynthesis. We speculate that ZjSWEET2.2 indirectly improves photosynthesis by stimulating phloem loading and decreasing the carbohydrate levels in mesophyll cells.

### Sugar Signals Mediate Expression of *ZjSWEET2.2*

Accumulation of free sugars can lead to down-regulation of photosynthesis through sugar signaling networks (Rolland et al., 2006). In this study, we found that the abundance of *ZjSWEET2.2* transcripts was significantly negatively correlated with sugar accumulation in fruits, and was significantly reduced by exogenous sugars at a high concentration. These results suggested that high concentrations of soluble sugars may also down-regulate the expression of the sugar exporter ZjSWEET2.2. In contrast, a low concentration of exogenous glucose promoted *ZjSWEET2.2* expression, indicating that it is induced by low-sugar signals. Both sugar-induction and sugar-repression *cis*-acting regions were identified in the promoter of *ZjSWEET2.2*, including three W boxes (TTGACC/T), which are indispensable for mediating sugar signaling (Sakr et al., 2018). The WRKY-type transcription factors specifically recognize W-Box *cis*-elements (Eulgem et al., 2000; Jiang et al., 2017a) and are known to be involved in sugar induction of gene expression (Chen et al., 2019). However, we detected more sugar-repression elements than sugar-induction elements in the promoter of *ZjSWEET2.2*. A previous study demonstrated that sugar regulatory motifs in the promoter of *STP1* in *Arabidopsis* are involved in repression of gene expression by glucose (Cordoba et al., 2015). Six out of the seven different motifs identified in the promoter of *STP1* were also detected in the promoter of *ZjSWEET2.2.* We detected two TATCCA elements in the 2-kb region of the *ZjSWEET2.2* promoter. In rice, the TATCCAOSAMY motif is recognized by the MYB-type transcription factor OsMYBS2, which regulates expression of α-*Amy3* in response to sugar signaling (Lu et al., 2002; Baena-Gonzalez et al., 2007). Consistent with this, we speculate that some transcription factors bind to the *cis*-acting regions in the *ZjSWEET2.2* promoter to mediate its expression. Further research is needed to clarity the regulation mechanism of these putative orthologs.

### Model of Regulation Mechanism of *ZjSWEET2.2*

**Figure 6:**
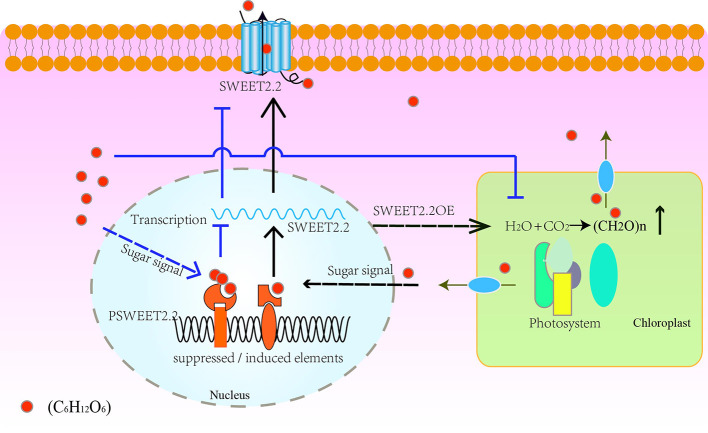
Schematic model of regulation of *ZjSWEET2.2* expression by sugar signals to mediate sugar transport. *ZjSWEET2.2* exports photosynthates from leaves under normal conditions. Over-expression of *ZjSWEET2.2* facilitates carbon fixation. Low sugar signals bind to inducing cis-elements in promoter of *ZjSWEET2.2* to activate its transcription. High sugar signals decreases the *ZjSWEET2.2* expression and reduces the photosynthetic rate.

On the basis of the expression patterns of *ZjSWEET2.2* in different tissues, we propose a hypothetical model for its regulation mechanism. As shown in **Figure 6**, our results suggest that *ZjSWEET2.2* plays a critical role in exporting sugars from leaves. Low sugar content may promote *ZjSWEET2.2* expression. Over-expression of *ZjSWEET2.2* increases carbon fixation into photosynthates by decreasing the carbohydrate content in mesophyll cells. When the sugar content increases in the cytosol, such as under drought stress or in ripening fruit, the expression of sugar-responsive transcription factors increases. These bind to sugar-suppressed elements in the promoter region of *ZjSWEET2.2* to repress its expression. Besides, the over-accumulation of sugar reduces the photosynthetic rate (Baker et al., 2016). This model also explains the phenomenon of high expression levels of *ZjSWEET2.2* in mature leaves, but decreasing levels in ripening fruit as sugars accumulate.

## Data Availability Statement

The datasets presented in this study can be found in online repositories. The names of the repository/repositories and accession number(s) can be found in the article/**Supplementary Material**.

## Author Contributions

YG was responsible for the part of over-expression of SWEET2a and incubated jujube seedlings. MW measured the sugar content of jujube. CZ was accountable for design of the work, analysis, drafting the work.

## Funding

This work was supported by the Chinese National Natural Science Foundation (Grant no. 31800573) and The National Key Research and Development Program of China (2018YFD1000607).

## Conflict of Interest

The authors declare that the research was conducted in the absence of any commercial or financial relationships that could be construed as a potential conflict of interest.

## Abbreviations

*SWEET2a*, sugars will eventually be exported transporter 2a; *RBCS*, ribulose bisphosphate carboxylase/oxygenase; *PGK*, phosphoglycerate kinase; *RPI*, ribose-5-phosphate isomerase; *PRK*, phosphoribulokinase.
