# Supplementary material for: Sugar Transporter ZjSWEET2.2 Mediates Sugar Loading in Leaves of Ziziphus jujuba Mill
Source: Front Plant Sci. 2020 Jul 24;11:1081. doi: 10.3389/fpls.2020.01081 (PMC7396580; doi:10.3389/fpls.2020.01081)
Supplement: Supplementary file 1 [file DataSheet_1.docx]

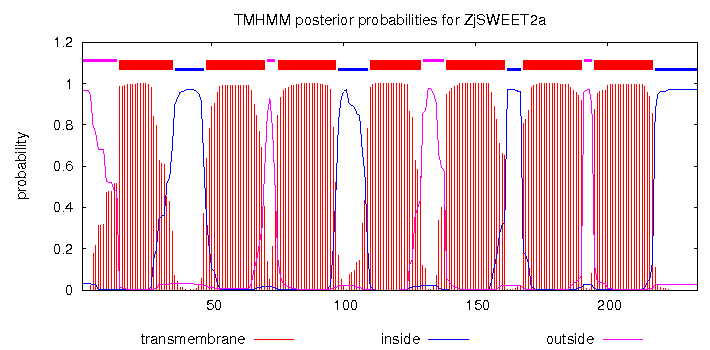


Supplemental figure 1 Predicted transmembrane domains in ZjSWEET2.2.

**Supplemental figure 2 The 2kp upstream promoter sequences of ZjSWEET2.2. Sequences marked with different color represent different cis-acting elements involved in sugar sigarnal.**


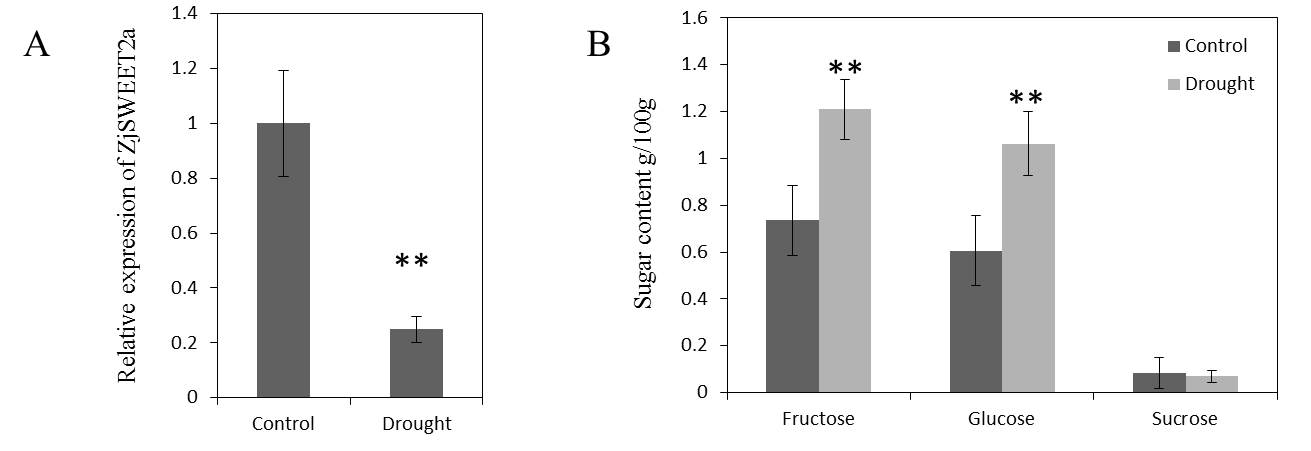


**Supplemental figure 3 Transcript levels of *ZjSWEET2.2* and sugar contents in leaves of jujube plants under drought stress. Three biological replicates were analyzed. Error bars represent standard error (SE). Asterisks indicate significant difference (Student’s t-test, ***P* < 0.01; **P* < 0.05).**

Supplemental table 1 Primer sequences and amplication characterristics of carbon fixation genes for RT-qPCR analysis.

| geneID | Primer 5'to 3' | Tm (℃） |
| --- | --- | --- |
| LOC107412938F | TCTGTCACCGCTACCAACAT | 56 |
| LOC107412938R | AAAGGACTGCCATACCAAAA | 56 |
| LOC107417353F | ATACCGTGAGAACCACAGATC | 56 |
| LOC107417353R | GAGGCTTGTAAGCAATGAAA | 56 |
| LOC107417243F | TACCGTGAGAACCACAGATC | 56 |
| LOC107417243R | GAGGCTTGTAAGCAATGAAA | 56 |
| LOC107403244F | TCCGATCAACAACACGAAGT | 56 |
| LOC107403244R | TGCGACTATTCTCCCTGTGC | 56 |
| LOC107429454F | TTACTACGATGGACGCTACTGG | 56 |
| LOC107429454R | ATACGGATGAAGGCATTTGG | 56 |
| LOC107434568F | GCCCGATTTCGATGCTTATA | 56 |
| LOC107434568R | CAGACACCTCGTGACCGTAG | 56 |
| LOC107412211F | CGGGCTTGTTCCTCTGTTCGTTTT | 56 |
| LOC107412211R | TCCATCACCGCTCACTCTGC | 56 |
| LOC107418684F | ACCGCTATCATTGGTCGTCG | 56 |
| LOC107418684R | ATGCCCTATTGCTGGTCTCC | 56 |
| LOC107429360F | TCCGATCTCGATTCCCACCC | 56 |
| LOC107429360R | AACCGGCATAGCCAAACCAC | 56 |
| LOC107422427F | AAAGGGCAGTGGAGCTACTCAT | 56 |
| LOC107422427R | GGCAGACCGAATCTCAGGAA | 56 |
| LOC107433843F | ACCGCTATCATTGGTCGTCG | 56 |
| LOC107433843R | ATGCCCTATTGCTGGTCTCC | 56 |
| LOC107417915F | GGAAGAGGGTATTTGTTAGAGT | 56 |
| LOC107417915R | TCAAGTATCGGATGGTAGGC | 56 |
| LOC107417916F | AAGGCACAAGGTCTTTCAGT | 56 |
| LOC107417916R | GTCCAATATCCAATCCCATC | 56 |
| LOC107431911F | CTGCAAAGGTCGTTAATAGA | 56 |
| LOC107431911R | CATTACTCCAAATGGGTTCT | 56 |

Supplemental table 2 Summary of the SWEET genes identified in *Ziziphus jujuba*, *Arabidopsis*, and *Solanum lycopersicum* L. plants.

| Gene | Genename | Gene | Genename | Gene | Genename |
| --- | --- | --- | --- | --- | --- |
| Zj.jz044739008 | ZjSWEET1 | At1g21460.1 | AtSWEET1 | Solyc04g064610 | SlSWEET1.1 |
| Zj.jz044815042 | ZjSWEET2.1 | At3g14770.1 | AtSWEET2 | Solyc04g064620 | SlSWEET1.2 |
| Zj.jz042571026 | ZjSWEET2.2 | At5g53190.1 | AtSWEET3 | Solyc04g064630 | SlSWEET1.3 |
| Zj.jz036789032 | ZjSWEET3 | At5g62850.1 | AtSWEET4 | Solyc04g064640 | SlSWEET1.4 |
| Zj.jz002249010 | ZjSWEET4.2 | At5g62850.1 | AtSWEET5 | Solyc06g060590 | SlSWEET1.5 |
| Zj.jz002249011 | ZjSWEET4.3 | At1g66770.1 | AtSWEET6 | Solyc06g060580 | SlSWEET1.6 |
| Zj.jz029235035 | ZjSWEET4.4 | At4g10850.1 | AtSWEET7 | Solyc02g071520 | SlSWEET2.1 |
| Zj.jz040715021 | ZjSWEET5 | At5g40260.1 | AtSWEET8 | Solyc07g062120 | SlSWEET2.2 |
| Zj.jz040945111 | ZjSWEET7 | At2g39060.1 | AtSWEET9 | Solyc03g007360 | SlSWEET3 |
| Zj.jz001627113 | ZjSWEET9.1 | At5g50790.1 | AtSWEET10 | Solyc03g114200 | SlSWEET5.1 |
| Zj.jz034227050 | ZjSWEET9.2 | At3g48740.1 | AtSWEET11 | Solyc02g086920 | SlSWEET6.1 |
| Zj.jz019271069 | ZjSWEET10.1 | At5g23660.1 | AtSWEET12 | Solyc08g082770 | SlSWEET7.1 |
| Zj.jz035233003 | ZjSWEET10.2 | At5g50800.1 | AtSWEET13 | Solyc12g055870 | SlSWEET7.2 |
| Zj.jz044343036 | ZjSWEET10.3 | At4g25010.1 | AtSWEET14 | Solyc03g097580 | SlSWEET10.1 |
| Zj.jz044343040 | ZjSWEET10.4 | At5g13170.1 | AtSWEET15 | Solyc03g097600 | SlSWEET10.2 |
| Zj.jz015029070 | ZjSWEET11 | At3g16690.1 | AtSWEET16 | Solyc03g097610 | SlSWEET10.3 |
| Zj.jz042065052 | ZjSWEET13 | At4g15920.1 | AtSWEET17 | Solyc03g097870 | SlSWEET11.1 |
| Zj.jz031515072 | ZjSWEET16 |  |  | Solyc03g097570 | SlSWEET11.2 |
| Zj.jz031515064 | ZjSWEET17.1 |  |  | Solyc06g072620 | SlSWEET11.3 |
| Zj.jz034557032 | ZjSWEET17.2 |  |  | Solyc06g072640 | SlSWEET11.4 |
|  |  |  |  | Solyc03g097590 | SlSWEET12.1 |
|  |  |  |  | Solyc03g097620 | SlSWEET12.2 |
|  |  |  |  | Solyc05g024260 | SlSWEET12.3 |
|  |  |  |  | Solyc06g072630 | SlSWEET12.4 |
|  |  |  |  | Solyc03g097560 | SlSWEET14 |
|  |  |  |  | Solyc01g099880 | SlSWEET16 |
|  |  |  |  | Solyc01g099870 | SlSWEET17 |
